# Lipid metabolic reprogramming as an emerging mechanism of resistance to kinase inhibitors in breast cancer

**DOI:** 10.20517/cdr.2019.100

**Published:** 2020-03-19

**Authors:** William W. Feng, Manabu Kurokawa

**Affiliations:** 1 Department of Molecular and Systems Biology, Geisel School of Medicine at Dartmouth, Hanover, NH 03755, USA.; 2 Department of Biological Sciences, Kent State University, Kent, OH 44242, USA.

**Keywords:** Drug resistance, lipid metabolism, small molecule inhibitor, tyrosine kinase, HER2

## Abstract

Breast cancer is one of the leading causes of death in women in the United States. In general, patients with breast cancer undergo surgical resection of the tumor and/or receive drug treatment to kill or suppress the growth of cancer cells. In this regard, small molecule kinase inhibitors serve as an important class of drugs used in clinical and research settings. However, the development of resistance to these compounds, in particular HER2 and CDK4/6 inhibitors, often limits durable clinical responses to therapy. Emerging evidence indicates that PI3K/AKT/mTOR pathway hyperactivation is one of the most prominent mechanisms of resistance to many small molecule inhibitors as it bypasses upstream growth factor receptor inhibition. Importantly, the PI3K/AKT/mTOR pathway also plays a pertinent role in regulating various aspects of cancer metabolism. Recent studies from our lab and others have demonstrated that altered lipid metabolism mediates the development of acquired drug resistance to HER2-targeted therapies in breast cancer, raising an interesting link between reprogrammed kinase signaling and lipid metabolism. It appears that, upon development of resistance to HER2 inhibitors, breast cancer cells rewire lipid metabolism to somehow circumvent the inhibition of kinase signaling. Here, we review various mechanisms of resistance observed for kinase inhibitors and discuss lipid metabolism as a potential therapeutic target to overcome acquired drug resistance.

## INTRODUCTION

Following the breakthrough success of Gleevec, small molecule kinase inhibitors have become a paradigm-shifting class of cancer therapeutics over the past two decades. As opposed to function blocking antibodies, kinase inhibitors are small molecules permeable to the blood-brain barrier and are therefore more efficacious in treating brain tumors. Furthermore, the ability to selectively target oncoproteins of interest offers a clinical benefit in mitigating off-target toxicities often seen with radiation therapy and conventional chemotherapy, such as doxorubicin and cisplatin. In the pre-clinical research setting, kinase inhibitors serve as valuable tool compounds that are able to rapidly inhibit kinase activity in tissue culture cell lines across species. Moreover, kinase inhibitors allow researchers to dissociate the role of kinase activity from protein scaffolding, a distinct advantage over RNAi- and CRISPR-mediated approaches. It is estimated that 25% of all ongoing drug development is dedicated to this class of drugs^[1]^. Since 2001, over 10,000 patent applications have been filed in the U.S. alone for kinase inhibitors^[1]^. As of June 2019, the FDA has approved 48 kinase inhibitors, 43 of which are indicated for the treatment of various cancers^[2]^. As ubiquitous and efficacious as these drugs have been, the common problem that continues to plague the clinic is the development of acquired drug resistance. Common mechanisms of resistance to kinase inhibitor resistance include kinase reprogramming^[3,4]^, overexpression of drug efflux pumps^[5]^, and drug resistant mutations^[6]^ among others.

Breast cancer is the most commonly diagnosed malignancy in women worldwide, with an estimated incidence of nearly two million cases annually^[7]^. Breast cancers can be categorized into three molecular subtypes based on expression of hormone receptors (HR), such as estrogen receptor (ER) or progesterone receptor (PR), and amplification/overexpression of the human epidermal growth factor receptor 2 (HER2). Roughly 70% breast cancers fall into the ER^+^ category, 20% fall into the HER2^+^ category, and the remaining 10%, which do not exhibit ER/PR expression or HER2 amplification/overexpression, are referred to as triple negative. Molecular subtype is the major determining factor which guides treatment strategies in breast cancer. As with other cancer types, treatment regimens often include kinase inhibitors. The HER2 receptor tyrosine kinase and the cell cycle regulators cyclin-dependent kinases 4 and 6 (CDK4/6) are targeted in HER2^+^ and ER^+^ breast cancer, respectively. However, development of drug resistance often limits therapeutic efficacy of these drugs to transient clinical benefits. Mechanisms of resistance to these inhibitors often revolve around PI3K/AKT/mTOR pathway hyperactivation and targeting this compensatory pathway has been shown to lead to re-sensitization^[8-11]^.

In this review, we highlight altered lipid metabolism as an emerging mechanism of resistance to small molecule kinase inhibitor therapy in breast cancer cells. Lipid metabolic rewiring is becoming increasingly recognized as a critical mechanism that promotes the growth and survival of cancer cells^[12-15]^ and is often associated with maintenance of cancer cell stemness^[16,17]^ and development of chemoresistance^[17,18]^. It is becoming clear that resistance mechanisms to kinase inhibitors in breast cancer often involve PI3K/AKT/mTOR pathway hyperactivation^[8-11,19]^. Notably, PI3K/AKT/mTOR pathway plays a critical role in the regulation of several lipid metabolic processes such as FA synthesis^[20]^, FA uptake^[21]^, FA storage into lipid droplets^[22,23]^ and FAO ^[24,25]^. Recent reports have demonstrated that targeting lipid metabolic pathways may be a valuable therapeutic strategy to re-sensitize cells resistant to HER2-targeted therapies in breast cancer^[18,26,27]^. We discuss the rationale behind targeting lipid metabolic rewiring in the treatment of drug resistant breast cancer cells as a therapeutic approach that requires further investigation.

## KINASES TARGETED IN BREAST CANCER

### HER family receptor tyrosine kinases in HER2^+^ breast cancer

Twenty percent of breast cancers exhibit overexpression or genetic amplification of the HER2 receptor and are classified as the HER2^+^ subtype^[28]^. HER2 is a member of the HER family of receptor tyrosine kinases, which include EGFR (HER1), HER2, HER3, and HER4. Upon binding to exogenous ligands, these receptors undergo homo- and heterodimerization, transphosphorylation, and activate downstream PI3K/AKT and MAPK pathways to promote cancer cell growth and survival^[29]^. Unlike other HER family members, HER2 is constitutively active and does not require ligand-induced activation^[30]^. HER2 is always in an active conformation and is known to be the preferred dimeric partner of all HER family members, with a preference for HER2-HER3 heterodimer formation^[29,30]^.

Although standard of care for HER2^+^ breast cancers involves treatment regimens centered around the HER2-targeted antibody trastuzumab, there are several small molecule inhibitors used in the clinic that also target HER family receptors [Table 1]. Lapatinib, a dual inhibitor of EGFR and HER2, was the first small molecule inhibitor FDA approved for the treatment of advanced HER2^+^ breast cancer. Displaying promise in pre-clinical studies, lapatinib was shown to inhibit the growth of trastuzumab-resistant cancer cells^[31]^ and was shown to have additive effects with HER2-targeted antibodies^[32,33]^, offering hope for the treatment of breast cancer patients who previously relapsed on trastuzumab-based therapies. Although lapatinib exhibited only modest clinical success as a single agent in humans^[34]^, it has been shown to be effective in certain settings. Addition of lapatinib to capecitabine displayed increased efficacy over capecitabine alone in women who progressed after treatment with an anthracycline + taxane + trastuzumab based therapy^[35,36]^. In the neoadjuvant setting, addition of lapatinib to trastuzumab was also shown to elicit superior clinical responses as compared to trastuzumab alone^[37]^. Importantly, lapatinib also demonstrated clinical activity in HER2^+^ breast cancer patients with brain metastases^[38]^, offering a distinct advantage over trastuzumab in managing CNS metastatic disease.

Mechanisms of lapatinib resistance have been widely studied in the literature. Common mechanisms include HER3^[3]^ and HER4^[4,27]^ amplification, ligand-mediated HER family hyperactivation^[39,40]^, PI3K hyperactivation^[10,11,19]^, and metabolic reprogramming of glucose^[41,42]^ and glutamine^[43]^ utilization pathways. Additionally, many of these also manifest as mechanisms of resistance to trastuzumab as well^[44,45]^.

In 2017, the FDA approved a second HER family-targeted inhibitor, neratinib, an irreversible inhibitor of EGFR, HER2, and HER4. HER3 is not targeted due to the lack of intrinsic kinase activity. Importantly, neratinib therapy was not shown to be associated with an increased incidence of cardiotoxicity^[46]^ as opposed to trastuzumab. However, the major reported side effect is high grade nausea and diarrhea^[46]^. In 2019, neratinib was granted Orphan Drug Designation and its indication was expanded to include the treatment of breast cancer-related brain metastases. Mechanisms of resistance to neratinib are still under investigation but targeting Src^[47]^ and mTORC1^[48]^ have shown promise. In addition to lapatinib and neratinib, gefitinib and afatinib are two other FDA approved kinase inhibitors that have shown clinical efficacy in breast cancer but are currently used only for the treatment of non-small cell lung cancer (NSCLC)^[49,50]^. More recently, addition of a new pan-HER inhibitor pyrotinib to capecitabine demonstrated increased clinical efficacy as compared to lapatinib and capecitabine in HER2^+^ patients who have progressed on taxanes, anthracyclines, and/or trastuzumab (NCT02422199). Although the drug is already approved for use in China^[51]^, it currently remains in several Phase III clinical trials in the U.S. (NCT03980054, NCT03080805, NCT03863223, NCT02973737, and NCT03588091).

### CDK4/6 inhibitors in ER^+^ breast cancer

Dysregulation of the cell cycle is a well-known hallmark of cancer^[52]^. Progression through the cell cycle is mediated by the formation of several heteromeric complexes consisting of a cyclin family protein and a serine/threonine cyclin-dependent kinase (CDK). In particular, the Cyclin D1-CDK4/6 complex plays a critical role in the progression from G_1_/S transition, a checkpoint regulated by the retinoblastoma-associated protein (Rb1). Under normal circumstances, mitogenic signaling induces the expression of D-type cyclins, Cyclin D1, 2, and 3^[53]^. Cyclin D then binds and activates CDK4 or CDK6, resulting in the phospho-inactivation of the tumor suppressors, Rb1 and Rb1-like proteins p130 and p107. In the hypophosphorylated state, Rb1 represses the transcription of genes mediating cell cycle progression by binding to the transactivation domain of E2F. Upon phosphorylation by the CDK4/6-Cyclin D complex, Rb1 is inactivated, releases E2F, and allows for induction of genes controlling cell cycle progression (Cyclin E and Cyclin A), DNA replication (MCM7 and PCNA), and mitotic progression (Cyclin B1 and CDK1)^[54]^. Induction of E2F subsequently promotes the formation of a positive feedback loop by inducing expression of E-type cyclins, which mediate S phase transition and DNA synthesis^[55]^. Antagonizing CDK4/6 phosphorylation promotes the preservation of hypophosphorylated Rb, thereby restoring the inhibition of E2F-mediated induction of downstream target genes.

There are currently three FDA approved CDK4/6 targeting drugs on the market: palbociclib, ribociclib, and abemaciclib [Table 1]. In 2015, palbociclib became the first CDK4/6 inhibitor to be approved by the FDA and was granted accelerated approval in combination with the nonsteroidal aromatase inhibitor letrozole as first-line therapy for treating advanced ER^+^HER2^−^ breast cancer following results from the Phase II PALOMA-1 study (NCT00721409), which showed that the addition of palbociclib to letrozole therapy in advanced ER^+^HER2^−^ breast cancer nearly doubled progression free survival from 10.2 months to 20.2 months^[56,57]^. The Phase II PALOMA-2 study (NCT01740427) demonstrated similar findings, with the addition of palbociclib to letrozole extending median progression free survival from 14.5 months to 24.8 months in patients with advanced ER^+^HER2^−^ breast cancer^[58]^. In 2016, the FDA expanded the indication of palbociclib to include combination therapy with fulvestrant for the treatment of advanced ER^+^HER2^−^ breast cancer that progressed on endocrine therapy following results from the Phase III PALOMA-3 study (NCT01942135), which demonstrated that addition of palbociclib to fulvestrant for the treatment of ER^+^HER2^−^ metastatic breast cancer that progressed on endocrine therapy extended median progression free survival from 4.6 months to 9.5 months^[56,59]^. Since then, the FDA has approved two additional CDK4/6 inhibitors: ribociclib in 2017 and abemaciclib in 2018. The combination of a CDK4/6 inhibitor with an aromatase inhibitor is now standard of care as first-line therapy for the treatment of ER^+^HER2^−^ breast cancer in the U.S.

Although dispensable for normal development of mammary tissue, Cyclin D is essential for breast tumor development and progression, rendering breast cancer cells selectively sensitive to CDK4/6 kinase inhibitors. Indeed, CDK4/6 inhibition sensitized ER^+^ and HER2^+^ breast cancer cells to tamoxifen and trastuzumab, respectively^[60]^. Recent work has highlighted the tight coupling between the ER and the Cyclin D1-CDK4/6 pathways, thereby rendering ER^+^ breast cancers especially dependent upon CDK4/6 for proliferation^[60]^. Cyclin D1 expression has long been known to be elevated and predictive of poor clinical outcome in ER^+^ breast cancer^[61]^. ER is known to promote induction of Cyclin D1 expression^[62]^. Moreover, Cyclin D1 (but not D2 or D3) has been shown to directly interact with ER to promote its transcriptional activity, thereby further establishing Cyclin D1 dependency in hormone receptor positive breast cancer^[63]^. Lastly, CDK4/6 inhibitors were also shown to be effective in sensitizing *PIK3CA* mutant breast cancer cells to PI3K inhibitors, demonstrating efficacy in overcoming intrinsic and acquired resistance to PI3K inhibition^[64]^.

As with HER2-targeted therapies, activation of PI3K/AKT/mTOR signaling is becoming increasingly recognized as an important mechanism in the development of resistance to CDK4/6 inhibitors ^[56,65-68]^. Herrera-Abreu demonstrated that AKT phosphorylation is increased following chronic treatment with palbociclib and that PI3K/AKT signaling is responsible for sustaining E2F-induced expression of Cyclin E and CDK2^[65]^. Notably, antagonizing PI3K also inhibited downstream CDK2 activation, offering an additional mechanism of suppressing proliferation^[65]^. Indeed, PTEN loss was also demonstrated to mediate resistance to the CDK4/6 inhibitor ribociclib as well as PI3Kα inhibitors in the clinical setting^[69]^. Furthermore, the combination of ER, CDK4/6, and PI3K triple targeting demonstrated greater efficacy than single or double therapies in *in vitro* and *in vivo* models of ER^+^ breast cancer^[65,70]^. Notably, inhibiting Rb phosphorylation by antagonizing CDK4/6 was shown to induce AKT activation by releasing Rb-mediated mTORC2 suppression^[66]^ and facilitate metabolic rewiring via mTORC1 to promote glycolysis and oxidative metabolism in pancreatic ductal adenocarcinoma cells^[68]^. Therefore, it would be valuable to determine whether mTOR hyperactivation could also promote loss of sensitivity to CDK4/6-targeted therapy. As such, *in vitro* studies have also shown that mTORC1/2 inhibition can decrease Cyclin D1, Rb phosphorylation, and E2F-mediated transcription^[71]^. Additionally, ER, CDK4/6, and mTOR triple targeting has also yielded promising results in pre-clinical models^[71]^. It is believed that upregulation of Cyclin D underlies many of these mechanisms of resistance and, importantly, Cyclin D and Cyclin E upregulation can be blocked by PI3K/mTOR inhibition^[67,72]^. Additional mechanisms of CDK4/6 resistance include CDK6 amplification^[73]^ and Cyclin E overexpression/amplification^[65,72,74]^. Lastly, Rb null cells are inherently resistant to CDK4/6 inhibition^[75]^. Furthermore, Rb1 loss and mutations were shown to be associated with CDK4/6 inhibitor resistance in patient-derived xenograft (PDX) models^[65]^ and in patients with metastatic breast cancer^[69,76]^.

## ROLE OF PI3K/AKT/MTOR PATHWAY IN LIPID METABOLISM AND DRUG RESISTANCE

The PI3K family is composed of lipid kinases that phosphorylate the 3′OH group of phosphatidylinositols^[77]^. Class 1A PI3K family members are the most widely implicated in cancer and promote the conversion of phosphatidylinositol-4,5-bisphosphate (PIP2) on the plasma membrane to generate the second messenger, phosphatidylinositol-3,4,5-trisphosphate (PIP3)^[77]^. AKT and phosphoinositide-dependent protein kinase 1 (PDK1) directly bind to PIP3 and are recruited to the plasma membrane. PDK1 phosphorylation activates AKT and allows for activation of downstream target proteins that promote growth and survival in cancer cells. Mutation in *PIK3CA* is one of the most common events in breast cancer^[78,79]^ and hyperactivation of the PI3K/AKT pathway has long been thought to be a major downstream mechanism of resistance to HER2-targeted therapies^[3,80]^ as well as escape from hormone dependence in ER^+^ breast cancer^[81]^. While pharmacological inhibitors of PI3K and AKT have long been pursued as therapeutics in breast cancer, inhibitors have historically performed poorly in clinical trials due to off target effects/toxicity, such as autoimmune disorders, hypertension, dysregulation of blood glucose levels, and hepatotoxicity, among others^[82,83]^. However, in May 2019, alpelisib, a first in class PI3K inhibitor, was FDA approved for use in combination with fulvestrant for the treatment of men and postmenopausal women with HR^+^, HER2^−^, PIK3CA-mutated, advanced or metastatic breast cancer following progression on or after an endocrine-based regimen. Unfortunately, several pre-clinical models of PI3K inhibitors have revealed compensatory mechanisms of resistance, such as loss of PTEN^[84]^ or PI3K amplification or mutation^[85,86]^. Moreover, HER3 amplification can also occur, thereby facilitating PI3K hyperactivation as well^[87]^.

mTOR is the catalytic component of two functionally distinct protein complexes, mTOR complexes 1 (mTORC1) and 2 (mTORC2), whose activities are regulated by different cofactors. mTOR is a master regulator of cell growth and metabolism and is often dysregulated in a variety of cancers. mTOR regulates the expression and activity of several pertinent metabolic enzymes involved in amino acid, glucose, nucleotide, and lipid metabolism^[88]^. Furthermore, mTOR-mediated metabolic reprogramming has been shown to promote resistance to a variety of chemotherapies such as lapatinib^[9]^ and neratinib^[48]^. There currently are two mTOR inhibitors that are FDA approved. Temsirolimus was approved in 2007 for the treatment of advanced kidney cancer and everolimus was approved in 2012 for use in combination with the aromatase inhibitor exemestane for the treatment of HR^+^HER2^−^ metastatic breast cancer after progression on letrozole or anastrozole therapy. Everolimus is also indicated for a variety of other cancers such as kidney cancer, subependymal giant cell astrocytoma, and renal angiomyolipomas associated with tuberous sclerosis, as well as neuroendocrine tumors of pancreatic origin, gastrointestinal origin, or lung origin.

While the PI3K/AKT/mTOR pathway is extensively studied for its role in proliferative signaling in breast cancer, it is important to note that this central signaling axis also plays a critical role in regulating various aspects of metabolism including glycolysis^[24,88]^, glutaminolysis^[89]^, fatty acid (FA) synthesis^[20,90]^, and FA oxidation (FAO)^[24]^.

### Glucose and glutamine metabolism

PI3K/AKT/mTOR signaling promotes glycolysis in a number of ways, such as GLUT1- and GLUT4-mediated^[91,92]^ glucose uptake into cells. Notably, mTORC2 promotes feedforward activation of AKT, which further activates hexokinase 2 to promote the entry of glucose into the glycolytic cascade^[88]^. Moreover, mTOR signaling is also thought to induce GLUT1 and HK2 expression via activation of HIF1a and MYC^[88,93,94]^. Pyruvate, the final product of glycolysis, can then be converted to lactate or acetyl coA for use in the TCA cycle. An additional mechanism of feeding the TCA that is regulated by AKT/mTOR is glutamate anaplerosis^[89]^. Glutamate can be converted to glutamine, which can then be converted to the TCA intermediate α-ketoglutarate (α-KG). Importantly, α-KG can have two distinct metabolic fates in the cell. α-KG can either be used as fuel for the TCA cycle or converted back into citrate, which can then be exported from the mitochondria and converted to acetyl coA by ATP citrate lyase (ACLY)^[89]^. Acetyl coA can then be converted to malonyl coA by acetyl coA carboxylase 1 (ACC1) and these two coA products can be used as substrates for FA synthesis.

### FA synthesis

FAs are critical biomolecules utilized by all cells in membrane biogenesis, energy production, and signal transduction. Cancer cell growth and proliferation require an abundance of FAs to accommodate rapid membrane synthesis, a process classically thought to depend solely upon endogenous FA synthesis in cancer cells^[95,96]^. Interestingly, cancer cells almost universally exhibit a “lipogenic phenotype” characterized by exacerbated levels of FA biogenesis, even in the presence of abundant circulating exogenous FAs^[95,97]^. It is estimated that ~90% FAs in cancer cells are synthesized by the enzyme FA synthase (FASN)^[95,98,99]^. FASN is known to be overexpressed across several cancer types, including breast cancer, with expression increasing with tumor stage and predictive of poor prognosis^[97,100-103]^. Importantly, under physiological conditions, normal cells exhibit a sole preference for acquiring FAs from the circulation rather than depending on *de novo* lipogenesis^[104]^. Furthermore, pharmacological inhibition of FASN has been shown to be lethal to cancer cells *in vitro* and *in vivo* but not to normal cells^[105]^, highlighting the importance of FAs in cancer biology while simultaneously revealing a promising therapeutic window. As such, FASN has been extensively investigated for use as a potential therapeutic target for decades.

FASN has emerged as an important enzyme in HER2^+^ breast cancers, in particular since HER2 directly phospho-activates FASN protein^[106]^ as well as regulating transcription of FASN mRNA^[107]^. FASN inhibitors have yielded promising results in animal studies[^108-110]^ but have achieved limited success in clinical trials due to poor pharmacokinetics^[111]^ and off target toxicities, such as anorexia^[112]^. To date, the only FASN inhibitor to advance to clinical studies is TVB-2640, which has been shown to elicit promising responses across a variety of tumor types, including KRAS^MUT^ NSCLC, ovarian, and breast cancers^[113]^. TVB-2640 is currently being investigated in a Phase II clinical study (NCT03179904), combining FASN inhibition with trastuzumab and paclitaxel for the treatment of late stage HER2^+^ breast cancers. Additionally, dysregulation of *de novo* lipogenesis can also involve overexpression of other FA synthesis pathway proteins, such as acetyl coA carboxylase (ACC) and ACLY^[114,115]^. Inhibitors of these proteins are also currently undergoing pre-clinical development^[116,117]^. Importantly, AKT has been shown to activate the master regulator of sterol regulatory element binding protein 1 (SREBP1) at the levels of gene expression and protein expression via mTORC1 signaling^[20,118,119]^. Activated SREBP1 is cleaved and acts as a transcription factor that induces the expression of a variety of FA synthesis genes, including *FASN*, *ACACA* (which encodes ACC1), *ACLY*, and *CD36*, which encodes a transmembrane FA uptake transporter.

### FA uptake

While the therapeutic efficacy of FASN synthesis has been largely studied, the role of deregulated FA uptake and β-oxidation pathways are gaining attention for their roles in promoting cancer cell growth and metastasis^[120-124]^. Indeed, the role of FA uptake and “opportunistic nutrient acquisition” has even been proposed as an emerging hallmark of cancer^[12]^. Recent work has shown that cancer cells are able to instruct neighboring adipocytes to release FAs, which in turn the cancer cell can acquire and utilize, implying a critical role for tumor-stromal crosstalk in the regulation of tumor growth^[120,121]^. Furthermore, it was reported that cancer cells can also acquire FAs from the circulation via lipoprotein lipolysis catalyzed by lipoprotein lipase (LPL)^[122]^. Importantly, LPL-mediated lipolysis of FAs requires FA channel-mediated uptake into cells. The canonical FA transporter downstream of LPL is CD36^[123]^. While CD36 (also known as platelet glycoprotein 4) is a multifunctional protein that regulates angiogenesis and cellular adhesion in addition to FA uptake, CD36 as a FA transporter has emerged as an important player in the progression of many cancer types^[124,125]^. Pascual *et al*.^[124]^ showed that inhibiting CD36 attenuated metastasis of oral squamous cell carcinoma^[124]^. Ladanyi *et al*.^[125]^ showed that adipocytes induce the expression of CD36 in ovarian cancer cells, thereby promoting metastasis^[125]^. Importantly, work from our lab has also identified a critical role of CD36 in the development of resistance to HER2-targeted therapies in breast cancer^[18]^. Wang *et al*.^[21]^ demonstrated that rapamycin attenuates murine hepatic steatosis by reducing CD36 expression via inhibiting transcriptional efficiency, offering a potential mechanism of reducing CD36 expression *in vivo*^[21]^. Overall, the role of FA uptake has been a previously understudied area that may have therapeutic potential and requires further investigation.

### FA β-oxidation

Cancer cells canonically exhibit preferential usage of the glycolysis pathway even in the presence of excess oxygen, a process referred to as the “Warburg Effect”^[126^]. However, cancer cells also often utilize the β-oxidative pathway to meet bioenergetics demands as well. In fact, β-oxidation is even known to be the dominant bioenergetic pathway in prostate cancer^[127]^ and has been demonstrated to be a critical metabolic pathway in triple negative breast cancer^[128,129]^. Furthermore, recent work has shown that FAO is a major source of acetyl coA for histone acetylation^[130]^ and that acidosis reprograms cancer cell FA metabolism via changes in histone acetylation^[131]^. These findings suggest that, in hypoxic regions of tumors where glycolysis likely fuels ATP production, cancer cells could adapt flexibility to use FAO to meet bioenergetic demands. Moreover, lipid droplet dynamics are known to play an essential role in regulating FA partitioning in cancer cells. Triglyceride (TG) synthesis and storage of excess FAs in lipid droplets protects cells from overabundance of free fatty acids and lipotoxicity. Interestingly, different breast cancer subtypes have been shown to exhibit distinct preferences in FA handling, such as the source of FAs used for TG synthesis. Although triple negative MDA-MB-231 cells exhibit greater levels of TGs as compared to ER^+^HER2^−^ MCF7 cells^[129]^, the former tend to divert exogenous FAs towards TG synthesis^[132]^ while the latter tend to rely upon *de novo* lipogenesis to support this process^[129]^. Two unique isoforms of diacylglycerol transferase (DGAT) mediate the synthesis of TGs in cells from FA and diacylglycerol, where DGAT1 promotes TGs synthesis from exogenous FAs and DGAT2 from endogenous FAs. As such, MDA-MB-231 cells express elevated levels of DGAT1, whereas MCF7 cells express elevated levels of DGAT2^[129]^. Lipid droplets are dynamic organelles and the TGs they carry can also be hydrolyzed by intracellular lipases to release FAs during times of need, such as nutrient stress. Notably, lipid droplets can serve as a pro-tumorigenic source of FAs. Indeed, monoacylglycerol lipase (MAGL) mediated FA lipolysis has been shown to promote migration, invasion, survival, and tumor growth^[133]^ and antagonizing MAGL was shown to attenuate this phenotype. Therefore, targeting FA esterification and lipid droplet hydrolysis have been proposed as potential therapeutic strategies that require further investigation^[133,134]^.

While the role of AKT/mTOR signaling on FA synthesis is well understood, its role in regulating β-oxidation is recognized but remains poorly characterized. mTOR inhibition by rapamycin was shown to reduced glucose transport capacity, glycogen synthesis, and glycolysis by approximately 40% but also induced β-oxidation by 60% in skeletal muscle cells^[24]^. In rat hepatocytes, rapamycin induced oxidation of exogenous FAs by 46%-100% and reduced esterification of exogenous FAs and *de novo* lipogenesis by 40%-60%^[25]^. Because rapamycin is a selective inhibitor for mTORC1, these effects are thought to be mediated by the mTORC1 complex.

## TARGETING LIPID METABOLIC REWIRING IN DRUG RESISTANT CANCER CELLS

**Figure 1.**
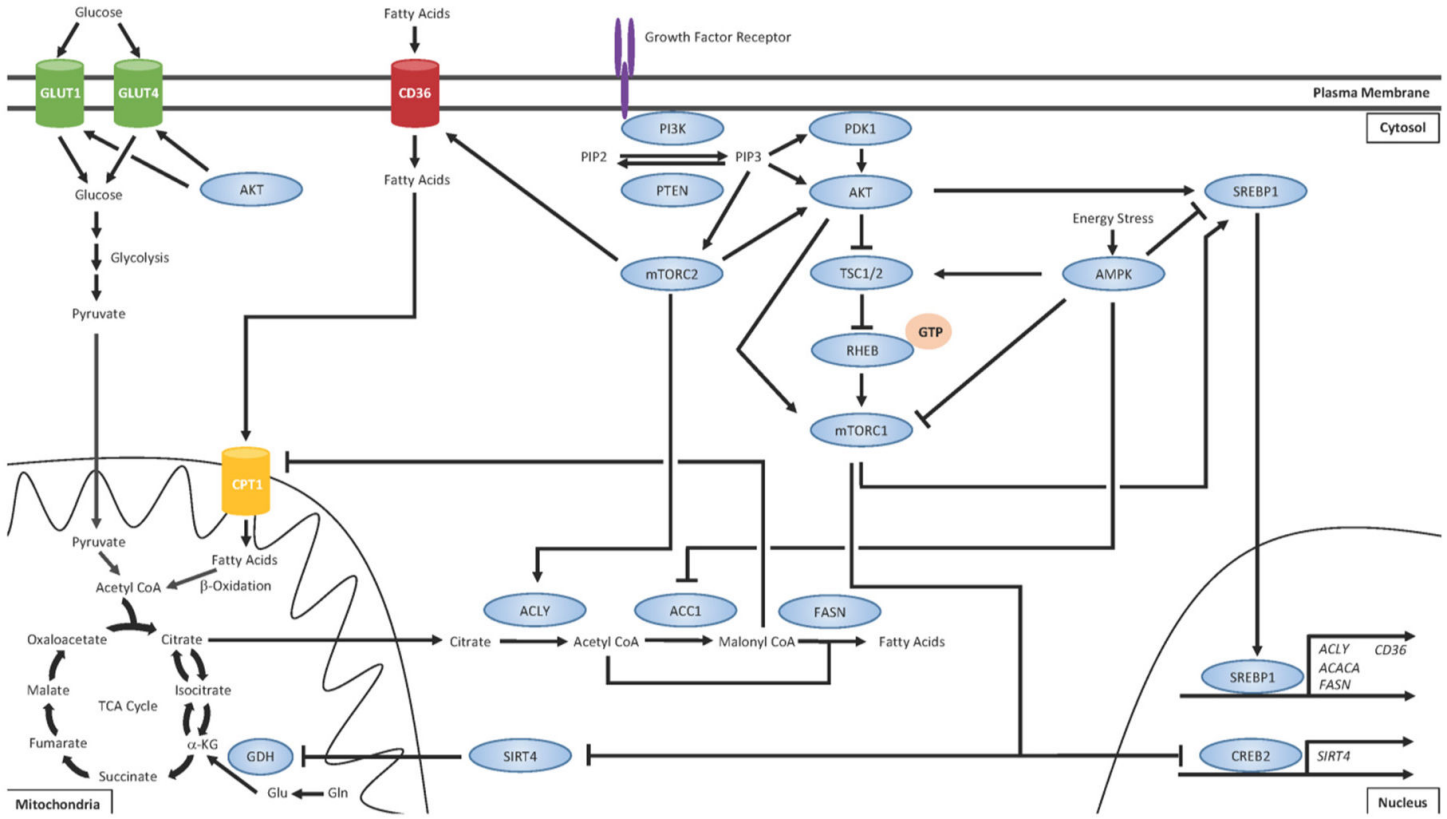
PI3K/AKT/mTOR regulates various aspects of lipid metabolism

Metabolic rewiring has long been recognized as a *bona fide* hallmark of cancer^[52]^ and the importance of lipid metabolism in cancer biology is gaining salience^[12-15]^. Apart from serving bioenergetics purposes, FAs exhibit a number of fates within cells including production of phospholipids for membrane biosynthesis and alteration of lipid raft composition to reconfigure growth factor receptor signaling^[95-135]^. Altered lipid metabolism in cancer cells has also been shown to promote growth and survival^[12-15]^, cancer cell stemness^[16,17]^, and chemoresistance^[17,18]^. Given the intricate crosstalk between the PI3K/AKT/mTOR pathway on regulation of FA uptake, synthesis, and oxidation, we believe that the hyperactivation of this pathway that often plays a driving role in the development of drug resistance may also coincide with concomitant changes in regulation of lipid metabolism [Figure 1].

Upon activation of growth factor receptors, such as the HER family receptor tyrosine kinases, the PI3K/AKT/mTOR signaling cascade is often the major effector pathway. The lipid kinase PI3K catalyzes the conversion of phosphatidylinositol-4,5-bisphosphate (PIP2) on the plasma membrane to generate the second messenger, phosphatidylinositol-3,4,5-trisphosphate (PIP3)^[77]^. PIP3 recruits phosphoinositide-dependent protein kinase 1 (PDK1) and AKT to the plasma membrane, where PDK1 activates AKT. Tuberous sclerosis complex 1 (TSC1) and TSC2 form a heterodimer and negatively regulate mTORC1. TSC2 possesses GTPase-activating protein activity and, under normal circumstances, stimulates RHEB GTPase activity to hydrolyze bound GTP and convert RHEB into its inactive state^[132]^. Activated AKT phosphorylates TSC2 and prevents TSC1/TSC2 heterodimerization, which allows for GTP loading onto RHEB^[132]^. GTP-bound RHEB can then directly activate mTORC1^[133,134]^. Furthermore, the mTORC1 component PRAS40 is also a direct substrate of AKT, allowing for an additional mechanism of mTOR pathway activation^[135]^. One of the major mechanisms of mTORC1-mediated regulation of lipid metabolism is the activation of the SREBP1 transcription factor, which promotes the transcription of several genes that promote FA synthesis, such as ACLY, ACACA (which encodes ACC1), and FASN, as well as FA uptake^[92,119]^. mTORC1 also stimulates glutamine (Gln) anaplerosis by activating glutamine dehydrogenase (GDH) via destabilizing CREB2 and suppressing expression of SIRT4^[89]^. Through this mechanism, Gln can be converted into glutamate (Glu) and then into α-KG. α-KG can then either be used as fuel for the TCA cycle or converted back into citrate, which can be exported from the mitochondria and converted to acetyl coA by ATP citrate lyase (ACLY). Acetyl coA can then be converted to malonyl coA by acetyl coA carboxylase 1 (ACC1) and these two coA products can be used as substrates for FA synthesis catalyzed by FASN. The mTORC2 complex serves several functions distinct from the mTORC1 complex. mTORC2 has been shown to activate AKT^[88]^ and ACLY^[136]^ as well as promote the expression of the FA uptake channel CD36^[137]^. In addition to regulating lipid metabolism, AKT can also promote membrane localization of GLUT transporters and increased glucose uptake and glycolysis^[91,92]^. Importantly, the master regulator of energy homeostasis AMP-activated protein kinase (AMPK) is well recognized as having opposing effects on mTOR signaling^[138-140]^. AMPK negatively regulates mTOR by phospho-inactivating TSC2 and the mTORC1 subunit Raptor^[141]^. While mTOR is generally active during conditions of high energy states, AMPK is activated during nutrient stress. AMPK restores energy balance by promoting signaling for catabolic processes such as glycolysis, autophagy, and FAO while concomitantly inhibiting energetically costly anabolic processes such as FA synthesis^[142]^.

Transcriptomic analysis of trastuzumab resistant HER2^+^ cell lines revealed genes involved in the lipid metabolism, glycolysis, and vitamin A metabolism as the most commonly differentially expressed as compared to parental cells, suggesting that metabolic reprogramming is associated with acquired resistance^[136]^. Several groups have also noted that endocrine resistance is associated with lipid metabolic reprogramming^[137,138]^. Hultsch *et al*.^[137]^ demonstrated that tamoxifen resistance is associated with increased lysosomal cholesterol accumulation^[137]^ and Du *et al*.^[138]^ demonstrated that MDA-MB-134 endocrine resistant cells exhibit exacerbated levels of SREBP1, the master regulator of FA, and cholesterol synthesis, and were hypersensitive to SREBP1 and FAO inhibition as compared to parental cells^[138]^. In addition, many mechanisms of resistance to CDK4/6 inhibitors, such as Rb loss^[65,69,76]^ and Cyclin D upregulation^[67]^, often result in increased activation of CDK4 signaling. Jin *et al*.^[139]^ identified C/EBPa as a critical target of the Cyclin D3-CDK4 complex^[139]^. C/EBPa is a major transcription factor that regulates adipocyte differentiation and controls the expression of genes such as CD36 and DGAT. Jin *et al*.^[139]^ found increased activation of Cdk4 in mouse models of nonalcoholic fatty liver disease (NAFLD) and human NAFLD patients^[139]^. They demonstrated that inhibition of Cdk4 can reverse hepatic steatosis in NAFLD mice, providing evidence that aberrant Cdk4 signaling can promote dysregulation of lipid metabolism in certain contexts^[139]^. Supporting this idea, Kitajima *et al*.^[140]^ demonstrated that Rb1 inactivation in MCF7 cells results in metabolic reprogramming and increased dependency on FAO^[140]^. Moreover, Franco *et al*.^[68]^ observed mTOR-mediated metabolic reprogramming in response to palbociclib treatment in pancreatic ductal adenocarcinoma cells that promoted increased glycolysis and oxidative metabolism ^[68]^. They observed that mTOR inhibition antagonized the metabolic changes and sensitized tumors to CDK4/6 inhibition^[68]^. Furthermore, Tarrado-Castellarnau *et al*.^[141]^ observed similar findings and identified MYC as a critical factor upstream of mTOR-mediated metabolic reprograming in response to CDK4/6 inhibition^[141]^. MYC was demonstrated to play a central role in modulating glutamine metabolism by upregulating glutamine transporters and transcriptionally repressing microRNA-23a/b to promote glutaminase (GLS1) expression^[141]^. GLS1 can promote mitochondrial respiration by converting glutamine to glutamate, which can then be converted to α-ketoglutarate (α-KG) and be used as fuel for the TCA cycle or as a substrate for *de novo* FA synthesis [Figure 1]. Importantly, GLS1 inhibition was shown to restore sensitivity to CDK4/6 inhibition^[141]^. Although the mechanism of lipid metabolic rewiring has yet to be fully elucidated in the arena of acquired resistance to CDK4/6 inhibitors, we encourage investigation into this area as we anticipate its importance. These observations raise the intriguing possibility of targeting lipid metabolism as an alternative therapeutic strategy in endocrine-resistant cells that have also stopped responding to CDK4/6 inhibition. Therefore, targeting these changes may allow for a promising therapeutic strategy to overcome acquired drug resistance to HER2-targeted and CDK4/6-targeted therapies in HER2^+^ and ER^+^ breast cancers, respectively.

Several groups recently demonstrated that breast cancer cells resistant to lapatinib and trastuzumab both exhibit increased sensitivity to FASN inhibition as opposed to parental cells and indicating that targeting FASN activity may be a valuable therapeutic approach to circumventing resistance to targeted therapy^[26,142]^. Additionally, it was also shown that FASN inhibition reduced HER2 levels in trastuzumab resistant cells and sensitized them to trastuzumab^[143]^. Lastly, Blancafort *et al*.^[27]^ found that the combination of anti-FASN polyphenolic compounds with an mTOR inhibitor (temsirolimus) and a HER2 dimerization domain-targeted antibody (pertuzumab) had the greatest combinatorial effect in suppressing the growth of lapatinib and trastuzumab dual resistant cells^[27]^.

Work from our lab has also identified CD36 as an important mediator of resistance to HER2-targeted therapies. We found that pharmacologic inhibition of CD36 with the small molecule inhibitor sulfosuccinimidyl oleate (SSO) or a function blocking antibody could re-sensitize lapatinib resistant cells to targeted therapy *in vitro* as well as *in vivo*^[18]^. Furthermore, RNA-seq analysis of human breast tumors pre- and post-treatment with trastuzumab and lapatinib (either alone or in combination) revealed that CD36 expression increased following treatment with targeted therapy. Furthermore, high CD36 expression exhibited by tumors of this cohort were also shown to be associated with poorer patient prognosis. Unfortunately, there are currently no FDA approved CD36 inhibitors that are available for use in humans. Therefore, it remains to be seen whether combination of CD36 inhibition can recapitulate the re-sensitization to HER2-targeted therapy observed in our preclinical studies.

Carnitine palmitoyl transferase (CPT1) is the rate limiting enzyme that catalyzes FAO. There are three isoforms of this enzyme, namely CPT1A, CPT1B, and CPT1C, which exhibit differences in tissue expression. All three isoforms have been reported to play an important role in the context of drug resistance. Samudio *et al*.^[144]^ showed that leukemia cells can exhibit mitochondrial uncoupling and promote a metabolic preference for FAO over pyruvate oxidation. Exploiting this dependency, they demonstrated that inhibition of carnitine palmitoyl transferase 1A (CPT1A) could sensitize leukemia cells to the BH3-mimetic ABT-747^[144]^. Wang *et al*.^[16]^ demonstrated that adipocyte derived leptin promotes JAK/STAT3 pathway activation in breast cancer cell to promote carnitine palmitoyl transferase B (CPT1B)-mediated dependency upon FAO and that this metabolic switch promotes cancer stemness and paclitaxel chemoresistance^[16]^. Importantly, inhibiting FAO re-sensitized resistant cells to chemotherapy^[16]^. Moreover, carnitine palmitoyl transferase 1C (CPT1C), the remaining isoform of CPT1, is commonly upregulated in lung tumors and promotes increased FAO, rapamycin resistance, as well as adaptation to metabolic stress such as glucose deprivation and hypoxia^[145]^. Inhibition of FAO by mercaptoacetate and etomoxir has also been shown to sensitize paclitaxel-resistant lung adenocarcinoma cells^[146]^. Lastly, Wu *et al*.^[147]^ identified long-chain fatty acyl coA synthetase (ACSL4), which facilitates FAO, as an important factor that promotes hormone and lapatinib resistance^[147]^. Therefore, several instances have demonstrated the therapeutic potential of targeting FAO to sensitize cancer cells to chemotherapies.

The energy sensor AMP-activated protein kinase (AMPK) is the master regulator of cellular energy homeostasis and is well recognized as having opposing effects on mTOR signaling by activating TSC2^[148-156]^. While mTOR is active during conditions of high energy states, AMPK is activated by elevated AMP:ATP ratio during nutrient stress and signals for catabolic pathways to restore energy balance^[157]^. AMPK negatively regulates mTOR by phospho-inactivating TSC2 and the mTORC1 subunit Raptor^[158]^. In doing so, AMPK promotes bioenergetics processes such as glycolysis, autophagy, and FAO, while concomitantly inhibiting energetically costly anabolic processes such as FA synthesis^[157]^. Importantly, palbociclib was recently shown to induce activation of AMPK^[159]^ and CDK4 has been shown to suppress FAO by directly antagonizing AMPKα2^[160]^. Therefore, AMPK activation may be a valuable therapeutic target to antagonize mTOR-mediated lipid metabolic reprogramming. The interconnectedness of signaling pathways that control lipid metabolism in cancer cells is summarized in Figure 1.

## CONCLUSION

Lipid metabolic reprogramming is an emerging mechanism of resistance to kinase inhibitor therapy in breast cancer. Given that mTOR hyperactivation is often implicated in development of acquired resistance to targeted therapy and that the AKT/mTOR signaling regulates various aspects of metabolism, exploiting rewired metabolic dependencies may allow for therapeutic re-sensitization of drug resistant cells. This would also allow for the possibility of repositioning existing lipid metabolic inhibitors into treatment regimens for breast cancer. Future work should be dedicated to identifying critically altered players in lipid metabolism that promote development of drug resistance and to assess whether targeting these pathways hold any therapeutic promise.

## Figures and Tables

**Table 1.** Kinase inhibitors for the treatment of breast cancer

Kinase inhibitor	Drug target	FDA approval status*
Lapatinib (Tykerb)	EGFR, HER2	Initially approved in 2007 and currently indicated for (1) use in combination with capecitabine for the treatment of patients with advanced or metastatic HER2+ breast cancer and who have received prior therapy including an anthracycline, a taxane, and trastuzumab or (2) in combination with letrozole for the treatment of postmenopausal women with ER^+^HER2^+^ for whom hormonal therapy is indicated
Neratinib (Nerlynx)	EGFR, HER2, HER4	Approved in 2017 for extended adjuvant treatment of early stage HER2^+^ breast cancer following adjuvant trastuzumab-based therapy and granted Orphan Drug Designation in 2019 for the treatment of breast cancer patients with brain metastases
Pyrotinib	EGFR, HER2, HER4	Approved for clinical use in China in combination with capecitabine for the treatment of advanced or metastatic HER2^+^ breast cancer. Currently in Phase III clinical trials in the U.S. (NCT03980054, NCT03080805, NCT03863223, NCT02973737, NCT03588091)
Palbociclib (Ibrance)	CDK4, CDK6	Granted accelerated approval in 2015 and currently indicated for the treatment of ER^−^HER2^+^ metastatic breast cancer in combination with (1) an aromatase inhibitor as initial endocrine-based therapy or (2) fulvestrant in patients who progressed on previous endocrine therapy
Ribociclib (Kisqali)	CDK4, CDK6	Approved in 2017 and currently indicated for the treatment of ER^−^HER2^+^ metastatic breast cancer in combination with (1) an aromatase inhibitor as initial endocrine-based therapy or (2) fulvestrant as initial endocrine-based therapy or following progression on endocrine therapy
Abemaciclib (Verzenio)	CDK4, CDK6	Granted priority review in 2015 and regular approval in 2017. Currently indicated for the treatment of ER^+^HER2^−^ metastatic breast cancer in combination with (1) an aromatase inhibitor as initial endocrine-based therapy or (2) fulvestrant following progression on endocrine therapy or (3) as monotherapy in patients exhibiting disease progression following endocrine therapy and prior chemotherpy in the metastatic setting
Alpelisib (Piqray)	PI3K	Approved in 2019 for use in combination with fulvestrant for the treatment of HR^+^HER2^−^ *PIK3CA* mutated advanced or metastatic breast cancer following progression on endocrine therapy
Everolimus (Afinitor)	mTOR	First approved in 2009 for the treatment of renal cell carcinoma and expanded to include treatment of ER^+^HER2^−^ metastatic breast cancer in combination with exemestane following progression on letrozole or anastrozole

* FDA approval statuses were found on the FDA website. Pyrotinib data referenced in^[31]^
